# The Role of Gut Microbiota in Lung Cancer: From Carcinogenesis to Immunotherapy

**DOI:** 10.3389/fonc.2021.720842

**Published:** 2021-08-19

**Authors:** Xiangjun Liu, Ye Cheng, Dan Zang, Min Zhang, Xiuhua Li, Dan Liu, Bing Gao, Huan Zhou, Jinzhe Sun, Xu Han, Meixi Lin, Jun Chen

**Affiliations:** ^1^Department of Oncology, The Second Hospital of Dalian Medical University, Dalian, China; ^2^Department of Oncology, The Third Hospital of Dalian Medical University, Dalian, China

**Keywords:** gut microbiota, lung cancer, immunotherapy, gut-lung axis, biomarker

## Abstract

The influence of microbiota on host health and disease has attracted adequate attention, and gut microbiota components and microbiota-derived metabolites affect host immune homeostasis locally and systematically. Some studies have found that gut dysbiosis, disturbance of the structure and function of the gut microbiome, disrupts pulmonary immune homeostasis, thus leading to increased disease susceptibility; the gut-lung axis is the primary cross-talk for this communication. Gut dysbiosis is involved in carcinogenesis and the progression of lung cancer through genotoxicity, systemic inflammation, and defective immunosurveillance. In addition, the gut microbiome harbors the potential to be a novel biomarker for predicting sensitivity and adverse reactions to immunotherapy in patients with lung cancer. Probiotics and fecal microbiota transplantation (FMT) can enhance the efficacy and depress the toxicity of immune checkpoint inhibitors by regulating the gut microbiota. Although current studies have found that gut microbiota closely participates in the development and immunotherapy of lung cancer, the mechanisms require further investigation. Therefore, this review aims to discuss the underlying mechanisms of gut microbiota influencing carcinogenesis and immunotherapy in lung cancer and to provide new strategies for governing gut microbiota to enhance the prevention and treatment of lung cancer.

## Introduction

The influence of gut microbiota on hosts has aroused intensive research interest. Microorganisms in the human gastrointestinal tract, counting more than 10 times the number of total host cells, with millions of whole, nonredundant genes, shape a dynamically balanced and healthy microenvironment within the body (1). A large number of studies have revealed that the gut microbiota is not only locally involved in the pathological process of gastrointestinal diseases [such as gastrointestinal tumors (2) and inflammatory bowel disease (3)] but also closely and systematically associated with nongastrointestinal diseases such as obesity (4), lung cancer (5), cardiovascular disease (6) and diabetes (7). It has been found that the gut microbiota affects host health homeostasis in many ways, and the gut microbiota and its related metabolites can disrupt the host’s normal cell cycle, leading to changes in cell and protein expression that control cell division, DNA repair, and apoptosis (8, 9). In addition, the gut microbiota is a major regulator of host inflammation and immunity, and it has been shown that the gut microbiota can affect host systemic inflammation and immune homeostasis, thereby increasing the susceptibility to malignant tumors and influencing the clinical immunotherapy response of tumors (10–12). Studies in animal models and humans have found that the gut microbiome is able to regulate the sensitivity of malignant solid tumors to immune checkpoint inhibitors (ICIs), such as programmed cell death receptor-1 (PD-1)/ligand 1 (L1) and cytotoxic T-lymphocyte-associated protein 4 (CTLA-4) (10, 13, 14).

Numerous studies have found that the gut microbiota is closely related to pulmonary pathology. Researchers found that the composition and function of the gut microbiota in patients with lung diseases [such as pneumonia (15), lung cancer (16), asthma (17), and tuberculosis (18)] were significantly changed compared with those in healthy people, and intervention with the gut microbiota can enhance the defense and efficacy of lung tissues against diseases (19, 20). Therefore, scientists have proposed the theory of the “gut-lung axis” to provide a reasonable explanation for the communication between the lung and gut (21, 22). Lung cancer is one of the most common malignant tumors. According to the latest statistics released by Globocan, the incidence of lung cancer is 11.4%, and the mortality rate is 18.0% in 2020; it has become the leading cause of cancer death (23). Because of the close relationship between the gut microbiota and lung, researchers have recently turned their attention to the gut microbiota for a new breakthrough in lung cancer prevention and treatment. Based on the current research, we speculate that the gut microbiota may not only be involved in the carcinogenesis of lung cancer, but can also affect the effectiveness of immunotherapy in lung cancer. Researchers found that the structure and function of the gut microbiota were unbalanced in patients with lung cancer, known as gut dysbiosis. Such an imbalance of *Firmicobacteria* and *Bacteroidetes* increased the risk of lung cancer (1, 16, 24), and the diversity of gut microbiota in patients with lung cancer was positively correlated with the efficacy of immunotherapy (25, 26). The interaction between gut microbiota and host may be attributed to its participation in host metabolism and immune functions. Therefore, a better understanding of the potential mechanisms by which the gut microbiota affects the occurrence and development of lung cancer as well as the response to immunotherapy is the key to forecasting the risk of lung cancer and improving the efficacy and safety of immunotherapy.

In this review, we summarized the existing research results to interpret how the composition and function of gut microbiota account for the carcinogenesis and immunotherapy of lung cancer. We will discuss how the gut microbiota regulates health and pathological immune responses through the gut-lung axis and how this regulation provides new ideas for effective and safe lung cancer prevention and treatment.

## Gut-Lung Axis

Emerging epidemiological and experimental evidence has highlighted a main intersection between the gut microbiota and the lungs, defined as the gut-lung axis (21). Although it has been shown that the gut-lung axis is a bidirectional communication channel, we mainly concentrate on the major direction of the cross-talk occurring from the gut to the lung in this paper. Numerous studies have shown that gut microbiota can not only affect lung homeostasis in a variety of ways, thus leading to increased susceptibility to lung diseases (21, 27, 28), but also enhance the resistance and recovery ability of the lung against diseases (29, 30). Changes in gut microorganism composition and metabolic function caused by the environment, diet, disease or medical intervention (such as antibiotics) are related to changes in the immune response and airway homeostasis (31, 32). Meanwhile, probiotics and fecal microbiota transplantation (FMT) could be used for the prevention and therapy of pulmonary diseases with widespread potential (33, 34).

**Figure 1:**
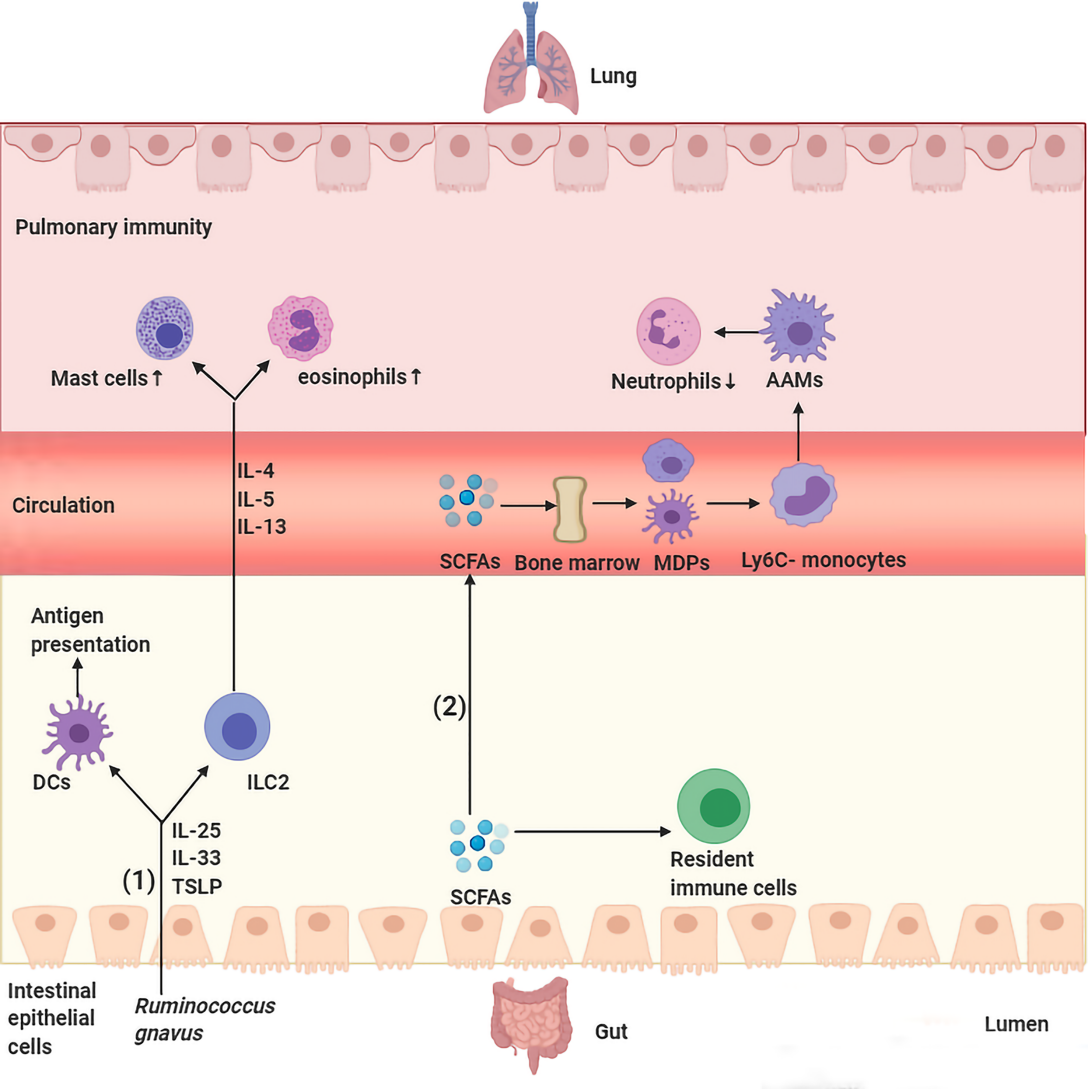
Major routes of communications within the gut–lung axis. (1) *Ruminococcus gnavus* stimulates secretion of IL-25, IL-33, and thymic stromal lymphopoietin (TSLP) by colon tissues, those cytokines activate DCs and ILC2 to produce cytokines IL-4, IL-5, and IL-13 and travel through the bloodstream to the lungs and lead to infiltration of the lung parenchyma by eosinophils and mast cells. (2) SCFAs transform macrophage and DC progenitors (MDPs) and their commitment into Ly6C− monocytes in the bone marrow, which can differentiate into alternatively activated macrophages (AAMs) in the lungs, thus control the immunopathology caused by infiltrating neutrophils.

Soluble microbial ingredients circulated *via* the gut-lung axis are one of the key interconnected manners by which gut microbiota participate in pulmonary diseases. Microbiota-derived antigens participate in the host immune response through the gut-lung axis and thus affect pulmonary immune homeostasis (**Figure 1**). Some studies have indicated that gut dysbiosis is common in patients with asthma, and dysregulation of specific bacterial taxa seems to be one of the powerful predictors of high asthma risk (17, 35–37). For example, researchers analyzed the gut microbiota of children with asthma and found that plasma metabolites (such as γ-tocopherol/β-tocopherol) and specific gut microbial taxa, such as the family *Christensenellaceae*, were positively related to asthma and asthma-associated intestinal derivatives (36). Similarly, Huey-Huey Chua et al. also found that before the appearance of allergic manifestations, overgrowth of *Ruminococcus gnavus (R. gnavus)* was discovered in children with allergic asthma, which means that *R. gnavus* could be a biomarker for airway allergies. In addition, mice presented with airway hyperresponsiveness and had histologic evidence of respiratory inflammation after treatment with purified *R. gnavus* (35). Mechanistically, augmentation of *R. gnavus* could stimulate the colon tissues to secrete cytokines [interleukin [IL]-33, IL-25, and thymic stromal lymphopoietin (TSLP)], with the result of activating dendritic cells and type 2 innate lymphoid cells to boost differentiation of T-helper 2 cells and production of their cytokines (IL-4, IL-5, and IL-13), which cause infiltration of the lung parenchyma by mast cells and eosinophils (35). In addition, changes in gut microbiota structure and function were also observed in patients with pulmonary tuberculosis (PTB). Investigators observed that tuberculosis patients show dramatic alterations in intestinal microbiota, as symbolized by striking decreases in microbial diversity and species populations (18, 38). The main manifestation is that PTB patients further presented upregulation of the opportunistic pathogen *Enterococcus* and the proinflammatory bacteria *Prevotella*, as well as a decrease in beneficial bacteria, including *Bifidobacteriaceae*, *Ruminococcaceae* and *Prausnitzii*, a significant reduction in short-chain fatty acid (SCFA)-producing bacteria as associated metabolic pathways, and a high pulmonary tuberculosis rate (18, 38). A study found that intestinal dysbiosis in mice caused by antibiotics decreased the expression of lung mincle (macrophage inducible C-type lectin) with subsequent increased survival of Mycobacterium tuberculosis (Mtb). Furthermore, antibiotics boosted regulatory T cell (Treg) numbers while restraining the frequency of effector and memory T cells in the lungs. Interestingly, administrating mice *Lactobacillus* resulted in normalization of the expression of mincle on pulmonary dendritic cells along with a concomitant anti-Mtb response (39). Of course, intestinal microbiota can not only lead to the occurrence of pulmonary diseases by interfering with pulmonary immune homeostasis but also protect pulmonary homeostasis to a certain extent. Tim J Schuijt et al. found that microbiota-depleted C57BL/6 mice infected with S. pneumoniae presented with higher bacterial dissemination, inflammation, organ damage and mortality than undepleted C57BL/6 mice. Interestingly, FMT to intestinal microbiota-exhausted mice miraculously rebounded lung bacterial counts and IL-10 and TNF-α levels (29).

In addition to recognizing the antigenic components of the intestinal flora, the host also senses microbiota-associated metabolites that are absorbed by the intestinal mucosa and then participate in pulmonary immunopathology along with blood and lymphatic circulation (**Figure 1**). The most typical example is SCFAs, including butyric, propionic and acetic acids, which are primarily sourced from the metabolism of dietary fiber in the colon and cecum. SCFAs regulate lung homeostasis and immunity by flowing into the body’s peripheral circulation and distal organs, such as the bone marrow, where they induce the differentiation of immune cells in the periphery with subsequent transportation to the lungs. For example, propionate or butyrate promotes the conversion of macrophages and DC progenitor cells (MDPs) into Ly6c- monocytes in the bone marrow, which are capable of differentiating into alternatively activated macrophages (AAMs) in the lungs, thus equipping them with anti-inflammatory and tissue repair abilities by controlling the immunopathology caused by infiltrating neutrophils (40, 41). SCFA treatment of dysbiotic mice regulated the activity of dendritic cells (DCs) and T cells and reduced the levels of circulating immunoglobulin E (IgE) and IL-4-producing CD4^+^ T cells, thus alleviating increased asthma susceptibility. Moreover, dendritic cells exposed to SCFAs less weakly activate T cells with lower responsiveness to CCL19 and present inhibited capacity to deliver inhaled allergens to pulmonary draining lymph nodes (42). Another study found that butyrate-producing gut bacteria can dampen lung group 2 innate lymphoid cell (ILC2) function, thus weakening the development of airway hyperreactivity in a mouse model (43). Furthermore, mice treated with SCFAs subsequently activate G protein-coupled receptor 43 (GPCR43), thus enhancing the capacity of macrophages to phagocytose invading Klebsiella pneumoniae (44). In addition to SCFAs, another metabolite that has also been shown to affect lung immune responses is bacterial-derived histamine; mice treated with *E. coli BL21_HTW*, bacteria that secrete histamine after genetic modification, showed decreased lung eosinophilia and depressed ovalbumin (OVA)-sensitized cytokine secretion from lung cells in an airway inflammation model (45). Other metabolites derived from gut microorganisms known to regulate immune homeostasis include indole derivatives (dietary tryptophan metabolites) (46, 47), niacin (45), polyamines (L-arginine metabolites) (48, 49), uridine A (50), pyruvate and lactic acid (51), all of which are thought to be important for intestinal homeostasis; however, whether these metabolites affect respiratory health still needs to be confirmed by numerous studies.

In general, the gut-lung axis is a well-connected cross-talk between the gut and the lungs, which is essential for shaping the immune system and maintaining host homeostasis, but the potential mechanisms through which the gut influences lung health or disease are only starting to be explored. We speculate that the gut microbiota and metabolites intruded into the intestinal mucosa are recognized by antigen-presenting cells and transferred to mesenteric lymph nodes (MLNs). They then activate immune cells and immune factors locally, which migrate through the lymphatic circulation and blood circulation and can act directly on lung target cells or continue to stimulate other immune cells. In addition, bacterial products or living bacteria from the gut can also travel through the blood or lymphatics to the lungs and stimulate the lung immune system. Thus, depending on tissue prestimulation, type of stimulation, and local and overall immune status, the gut-lung axis can effectively remove bacteria or anti-inflammatory activity, produce an excessive immune response, promote further tissue damage and disrupt pulmonary homeostasis (**Figure 1**).

## Characteristics of Gut Dysbiosis in Lung Cancer

Although few studies have been conducted on the characteristics of the intestinal flora of patients with lung cancer, similar acknowledgments have been achieved. The intestinal flora of patients with lung cancer is mainly characterized by significant changes in its composition and function, primarily manifested by a reduction in intestinal microbial diversity and a reduction in metabolic-related biological activities compared with healthy subjects (1, 16, 24, 52, 53). Many specific changes in intestinal bacterial composition (**Table 1**) and metabolism (**Table 2**) are closely related to lung cancer.

**Table 1:** Characteristic of composition in lung cancer patients.

Differential taxa features	Sample numbers	Sample type	References
Genera:	60	fecal specimens	He Zhuang et al. (1)
Enterococcus↑			
Actinobacteria↓			
Bifidobacterium↓			
Genera:	46	fecal specimens	Fang Liu et al. (24)
Bacteroides↑			
Prevotella↑			
Lachnospiraceae↓			
Genera:	181	fecal specimens	Yajuan Zheng et al. (16)
Ruminococcus↑			
Faecalibacteriu↓			
Bifidobacterium↓			
Veillonella↓			
Genera:	82	fecal specimens	Wei-Quan Zhang et al. (52)
Bacteroides↑			
Veillonella↑			
Fusobacterium↑			
Fecalibacterium↓			

↑, Microbiota increases in cases compared to controls; ↓, Microbiota decrease in cases compared to controls.

**Table 2:** Metabolic characteristics of gut microbiota in lung cancer.

Related metabolic pathway	Sample numbers	Sample type	References
RNA processing and modification↓	60	fecal specimens	He Zhuang et al. (1)
Chromatin structure and dynamics↓			
extracellular structures↑			
energy metabolism↓	46	fecal specimens	Fang Liu et al. (24)
ABC-type transport↓			
cellular antigens↑	181	fecal specimens	Yajuan Zheng et al. (16)
steroid biosynthesi↑			
ubiquitin system↑			
transcription-related proteins↑			
bile secretion↑			
fatty acid elongation in mitochondria↑			
bacterial motility proteins↓			
bacterial chemotaxi↓			
flavonol biosynthesis apoptosis and G protein-coupled receptors↓			
dodecane↑	19	fecal specimens	Pamela Vernocchi et al. (53)
2,6-dimethyl-4 heptanone methyl			
isobutyl ketone↑			
aldehydes↓			
ketones↓			
terpenes ↓			
p-cresol↓			

↑, Metabolism increases in cases compared to controls; ↓, Metabolism decrease in cases compared to controls.

Detection of the biological characteristics of the gut microbiome may be one of the promising methods for early-screening and prediction of lung cancer in the future. Analysis of fecal metagenomes found that despite remarkable interindividual differences, some predominant genera exhibited dramatic varieties between lung cancer and health. Lung cancer patients had a lower abundance of *Escherichia-Shigella*, *Kluyvera*, *Fecalibacterium*, *Enterobacter*, and *Dialister* but a higher abundance of *Bacteroides*, *Veillonella*, and *Fusobacterium* than healthy controls (52). Diversity and biomarkers of the gut microbiota for lung cancer were analyzed using next-generation sequencing. Increased abundance of *Enterococcus* yet decreased levels of the bacterial phylum *Actinobacteria* and genus *Bifidobacterium* were found in lung cancer patients compared to controls; the composition (beta diversity) differed remarkably between patients and controls, while the microbial diversity (alpha diversity) showed insignificant decline in lung cancer patients (1). In addition, the functional composition of 24 intestinal microbiota metabolic pathways was significantly depressed in lung cancer *via* a functional predictive analysis by COG (cluster of ortholog genes) functional annotation, especially those that participated in RNA processing and modification and chromatin structure and dynamics (1). Similarly, an imbalanced microbial ecosystem was observed in lung cancer, reflected as the elimination, low density, and loss of bacterial diversity microbial community featuring higher abundances of special pathogen microbiomes, including *Prevotella*, *Enterobacteriaceae*, *Streptococcus*, and lower probiotic genera, such as *Lachnospiraceae*, *Bifidobacterium*, *Blautia* and *Coprococcus*, versus healthy objects despite the interindividual complexity and diversity of the bacterial structures at the family and genus levels (24). Pathway comparisons *via* COG and KEGG (Kyoto Encyclopedia of Genes and Genomes) demonstrated that the functional abundance spectrum was broadly similar in lung cancer compared to healthy controls, while the microbiome exhibited less frequency in pathways involved in ABC-type (ATP-binding cassette type) transport and energy metabolism in lung cancer patients (24). One recent study not only identified imperfections in intestinal microbial diversity and metabolic pathways in lung cancer patients but also found that the specific intestinal microbial signature may be associated with lung cancer subtypes and metastatic status. The different subtypes present with distinctive microbiome profiles, and several lung cancer-associated bacteria, including *Blautia obeum*, *Lactobacillus salivarius*, *Akkermansia muciniphila* and an uncharacterized genus of family *Coriobacteriaceae* were overgrown in only three metastatic patients (16).

In summary, we found that the common characteristics of the intestinal flora in lung cancer patients are the decreased numbers of probiotics, increased conditional pathogenic bacteria populations, increased abundance of the genus *Bacteroidetes* and decreased abundance of the genus *Firmicutes*, resulting in a lower ratio of *Firmicutes/Bacteroidetes*. The reduced ratio of *Firmicutes/Bacteroidetes* in the gastrointestinal tract may lead to decreased circulating SCFAs (54, 55), and SCFAs can not only be induced by lung cancer cell apoptosis and cell cycle arrest (56), but also play an important role in host immunity and systemic inflammation (55, 57). However, contradictory results have also been found in those studies. Wei-quan Zhang et al. found an increase in the abundance of the genus *Veillonella* in lung cancer patients, while Yajuan Zheng et al. showed a decrease in its abundance (16, 52). This phenomenon can be explained by the difficulty in determining whether the specific species or strains are involved in carcinogenesis. The content may be too small to produce significant pathological results on its own, and the same species may also play protective or harmful roles depending on their lived environment. Therefore, more large-scale animal models and clinical patient studies are needed, and a deeper understanding of how the gut microbiota influences lung cancer and the underlying mechanism is needed before it can be used as a lung cancer biomarker or incorporated into treatment.

## Possible Mechanisms of Gut Microbiota Causing Lung Cancer

### Metabolism-Related Genotoxicity

Metabolites produced by some intestinal microbiota may be genotoxic and can directly induce DNA damage of host cells or modulate the basic host cell signaling pathways involved in cell proliferation and apoptosis. These interactions may lead to genetic and epigenetic modifications, thus endowing protumoral genome instability, which is most often caused by bacterial protein toxins that trigger host cell double-strand DNA breaks (DSBs), such as cytolethal distending toxin (CDT) (58, 59), cytotoxic necrotizing factor (CNF) (60, 61) and colibactin (62, 63). For example, researchers found that the human intestinal bacterial genotoxin colibactin alkylates DNA and thus shows a carcinogenic effect (62). In addition, certain bacterial toxins, such as Bacteroides fragilis toxin, contribute to cancer development or progression by altering major cell signaling pathways involved in cell proliferation and cell death (64–66). Andrew C Goodwin et al. found that B. fragilis toxin could also augment spermine oxidase, a polyamine catabolic enzyme, and subsequently result in the creation of reactive oxygen species (ROS) and DNA damage (67). Furthermore, bacteria can accelerate host cell transformation through protein virulence factors such as avirulence protein A (AvrA) (68, 69) and Fusobacterium nucleatum adhesion A (FadA) (70).

### Chronic Systemic Inflammation

At present, many studies have shown that the intestinal flora is closely associated with many diseases featuring chronic systemic inflammation (71–73). Intestinal bacteria not only influence immune and inflammatory responses at the local mucosal level but also lead to chronic pulmonary inflammation *via* gut-lung axis communication (52, 74). Gut dysbiosis can lead to damage to intestinal mucosal barrier function and increase intestinal mucosal permeability (75, 76); invading microorganisms and metabolites can cause local and systematic inflammation. Chronic inflammation is doubtlessly related to the occurrence and development of lung cancer (77–79). Therefore, we speculate that the disturbance of intestinal microbes and metabolites may lead to chronic systemic inflammation and then participate in the occurrence and development of lung cancer.

**Figure 2:**
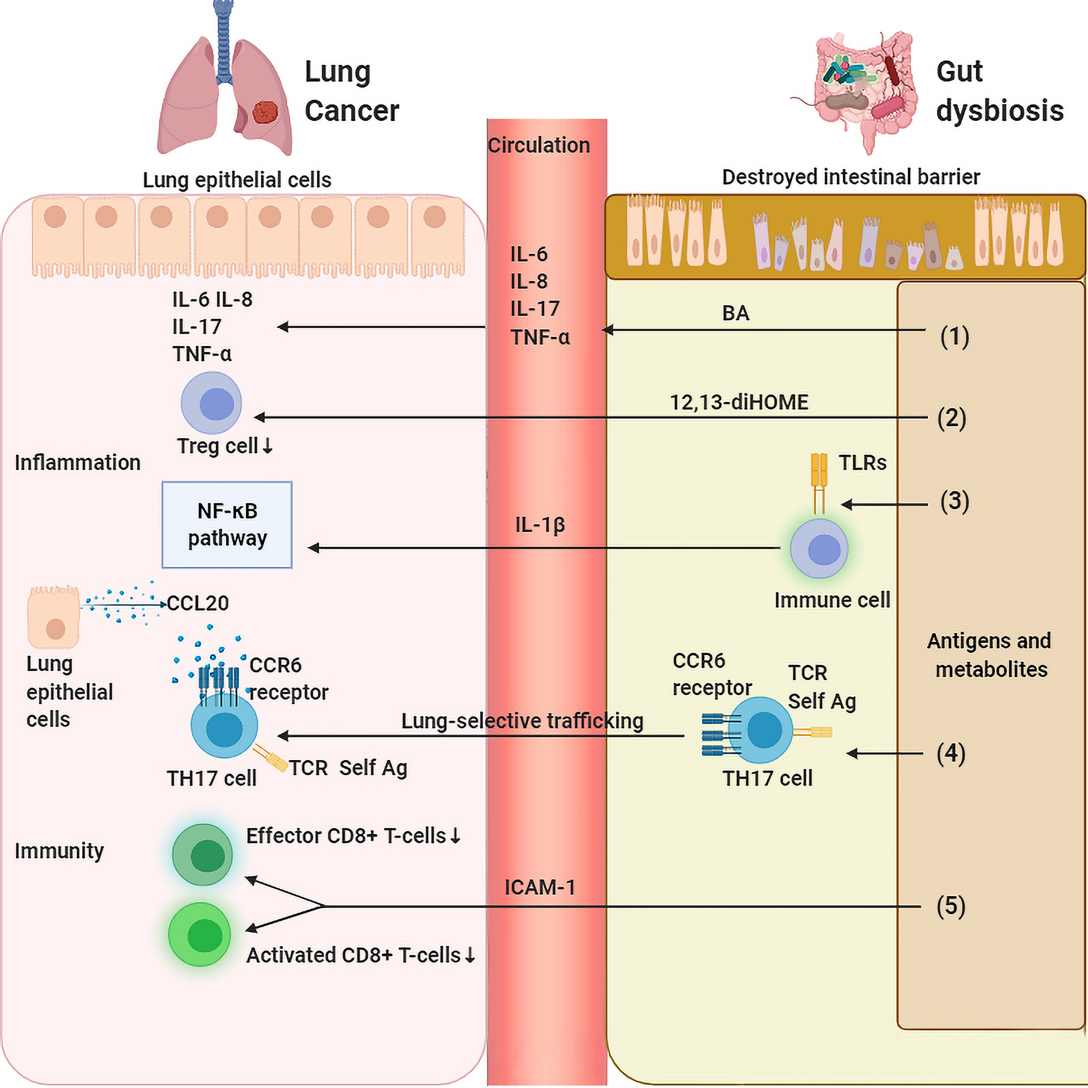
Gut dysbiosis regulates lung inflammation and immunity. (1) Gut dysbiosis causes damage to the intestinal mucosal barrier, invading gut bacteria and metabolites affect the host’s inflammation and immunity locally and systematically which in turn leads to the carcinogensis of lung cancer. Bile acid (BA) stimulates inflammatory markers such as IL-1β、IL-6 and IL-8 in the lung. (2) 12,13-diHOME decreases the number of regulatory T (Treg) cells in the lungs resulting in a reduced anti-inflammatory effect. (3) Bacteria-derived antigens activates TLR4 in the intestine immune cells, which increases the level of IL-1β in peripheral circulation that activates NF-κB pathway, leading to pulmonary inflammation. (4) SFB-induced gut Th17 cells are preferentially recruited to lung due to robust expression in the lung of CCL20. (5) Gut dysbiosis leads to a decrease in the expression of ICAM-1, which decreases the number of activated and effective CD8+ T cells in tumors.

The gut microbiota is an inexhaustible source of microbial-associated molecular patterns (MAMPs) and pathogen-associated molecular patterns (PAMPs) that can be recognized by nucleoside binding receptors (NODs) and Toll-like receptors (TLRs) on host cells. Direct contact with the lumen of TLRs exists not only in intestinal epithelial cells (IECs) but also in immune cells in the lamina propria, such as stromal cells, dendritic cells, macrophages, B cells and T cells. Microorganisms and their products entering the intestinal mucosa activate TLRs to produce inflammatory mediators and inflammatory factors, which participate in the pulmonary inflammatory process through lymphatic and blood circulation (**Figure 2**). For example, Stephen Wedgwood et al. found that gut dysbiosis characterized by a significant increase in *Enterobacteriaceae* activates TLR4 in the intestine and causes inflammation, increases the level of IL-1β in peripheral circulation, transduces inflammatory signals to the lungs, and activates the NF-κB pathway, leading to pulmonary inflammation (74). Similarly, Jia Tang et al. found that intestinal microbiota dysbiosis could modulate the TLR4/NF-kB signaling pathway in pulmonary immunity, subsequently motivating oxidative stress and inflammation to be involved in lung pathology by regulating the intestinal barrier (80).

In addition, some specific species have been shown to be closely related to the levels of systemic inflammatory factors in lung cancer. *Enterobacter* and *Escherichia-Shigella* were found to be significantly associated with serum neutrophil-to-lymphocyte ratio (NLR) levels, while *Dialister* was negatively associated with serum NLR and platelet-to-lymphocyte ratio (PLR) levels. Furthermore, serum CTLA-4 and IL-12 levels were correlated with *Dialister* (52). Jie Chen et al. found that the abundances of the genera *Enterococcus* and *Helicobacter* were strongly correlated with IL-6 levels (81). Another study found that enrichment of fecal microbial communities with the families *Lachnospiraceae* and *Ruminococcaceae* was correlated with increased concentrations of lung TNF-α and IL-17 (82). Antibiotic interventions in mice were found to lead to a significant decrease in the bacterial population and diversity, as well as a significant upregulation in the release of IL-6 in bronchoalveolar lavage fluid (BALF) (83).

Of course, gut microbiota-associated metabolites can also influence pulmonary inflammatory balance. Recent research has shown that human gut bacteria can produce other metabolites with proinflammatory potential, such as 12,13-diHOME (37) and bile acid (BA) (84). Mice intraperitoneally administered 12,13-DiHome presented with enhanced pulmonary inflammation and a suppressed population of Treg cells in the lungs, thus endowing them with a higher asthma susceptibility due to impedient immune tolerance, and 12,13-diHOME was capable of altering the expression of PPARγ-regulated genes of human dendritic cells and decreasing the secretion of anti-inflammatory cytokines and the count of Treg cells *in vitro* (37). In addition, the total BA concentration mainly produced by the gut microbiota was significantly associated with several inflammatory markers, such as IL-1β, IL-6 and IL-8, in bronchoalveolar lavage fluid (BALF) samples of cystic fibrosis patients (84). Of course, as mentioned above, intestinal flora metabolites represented by SCFAs also display powerful anti-inflammatory ability. However, a decrease in SCFA-producing bacteria and serum SCFAs is common in lung cancer patients, highlighting the complexity of gut microbiota-derived inflammation regulation.

In general, the gut microbiota, directly or indirectly through its metabolites and other components, actively participates in lung inflammation through microbial-cytokine regulatory interactions, both in the gut and systemically, leading to enhanced proinflammatory effects, weakened anti-inflammatory effects and chronic systemic inflammation, thus forming a microenvironment prone to the occurrence and development of lung cancer. Therefore, the regulation of intestinal flora will provide a new and promising treatment method for maintaining host homeostasis and preventing lung cancer.

### Immune Response

The intestinal microbiota is a significant factor contributing to the establishment of host pulmonary immune homeostasis, which affects the pulmonary immune response by regulating innate immunity and adaptive immunity. Gut dysbiosis can destroy the delicate balance between lung immune activation and immune tolerance, lead to tumorigenic inflammation due to excessive immune response, or lead to decreased antitumor ability due to defective immune surveillance function, thus forming a microenvironment conducive to the development of lung cancer cells (**Figure 2**).

Although we hypothesized that the antigenic components of gut microbiota may be transported to the lungs *via* the gut-lung axis and then directly participate in pulmonary immunity, few studies have been conducted in this area. The main mechanism is that the intestinal flora activates immunity in the intestine, and then these activated immune cells migrate to the lungs and participate in pulmonary immunity. However, how do intestinal immune cells migrate to the lungs through the gut-lung axis? This lung-selective trafficking of lymphocytes may be mainly due to chemokine-induced homing of lymphocytes (**Figure 2**). For example, Jerilyn Gray et al. found that intestinal congenital lymphocytes are closely related to pulmonary homeostasis, and intestinal flora mediates this tissue selective transport by increasing the high expression of the group 3 innate lymphoid cell (ILC2) homing receptor CCR4 but has no correlation with the proliferation or apoptosis of ILC3s (85). CCR4 is a chemokine receptor that is commonly identified as a biological signal for transporting T cells and Treg cells into the lungs (86). CCL17 is a ligand for CCR4 expressed on the lung epithelium with the capacity to activate the CCR4 receptor and promote ILC3 entry into the lungs (85). In addition, CD11c^+^ and CD8^+^ cells in the lungs of mice were found to be able to bind antigens from the intestinal microbiome. CD11c^+^ cells expressing CD8 are antigen-presenting cells equipped with the activity to shuttle between diverse tissues, and this effect may be related to the sufficient expression of TLR4 in CD11c^+^ cells in the lungs (87). In addition, C. Pierce Bradley et al. found that segmented filamentous bacteria (SFB) can induce pulmonary autoantibodies, and the Th17 cell response is necessary for SFB-dependent pulmonary pathology. SFB-induced intestinal Th17 cells are preferentially recruited to the pulmonary over spleen, accounting for strong expression in the lung of CCL20, the Th17 chemoattractant (88), and a ligand of CCR6 expressed on Th17 cells with the function of lymphatic tissue specific transportation. Studies have proven that it preferentially recruits Th17 cells to the inflammatory microenvironment of the central nervous system (89, 90). Additionally, dual T cell receptor (TCR)-expressing Th17 cells were selectively amplified by SFB, thus boosting autoimmune inflammation by identifying self-antigens and SFB epitopes in peripheral tissues (88).

In the case of immune response deficiency, Samir V Jenkins et al. showed that antibiotic-induced dysbiosis leads to faster progression of B16-F10 melanoma and Lewis lung cancer in mice. This progression is mediated by a decrease in local and systemic TNF-α levels, leading to a decrease in the expression of tumor endothelial adhesion molecules, especially intercellular adhesion molecule-1 (ICAM-1), and weakened leukocyte trafficking into the tumor, followed by a lower population of activated and effective CD8^+^T cells in tumors (91). In addition, Rodrigue Dessein et al. found that gut dysbiosis induces extensive cellular immunosuppression in the lung, reduces the circulating hematopoietic cytokine Fms-related tyrosine kinase 3 ligand (Flt3-Ligand), and suppresses dendritic cell bone marrow progenitors; pulmonary tissue manifested a noteworthy decline in macrophages, NKs, iNKT cells, γδ-T cells, cDC2, neutrophils and inflammatory monocytes due to extensive suppression of pulmonary cellular immunity by antibiotics under cell population analysis (92). Shikha Negi et al. found that intestinal microbiota imbalance decreased the expression of pulmonary macrophage inducible C-type lectin (mincle). In addition, antibiotics could reduce the number of effector and memory T cells (39).

## Immunotherapy

In recent years, immunotherapy has led to a new upsurge in tumor therapy. Many studies have investigated the intestinal flora as a novel biomarker that is closely related to the antitumor effects of ICIs, including CTLA-4 and PD-1/PD-L1 (10, 93–95). The diversity and stability of intestinal flora seem to be a biological signature for the sensitivity of anti-PD-1 immunotherapy in NSCLC patients, and some specific species seem to predict patients’ effectiveness to immunotherapy. In the Checkmete 078 and Checkmete 870 studies of 37 patients with advanced NSCLC treated with navumab, investigators found that patients who responded to PD-1 had higher intestinal microbiome diversity at the beginning of treatment, more stable intestinal microbiome composition during treatment, and significantly prolonged progression-free survival (PFS). The enrichment of *Bifidobacterium longum*, *Alistipes putredinis* and *Prevotella copri* was associated with better ICI efficacy (25). In addition, Peng Song et al. found that higher β-diversity in the intestinal flora of lung cancer patients treated with PD-1 blockade predicted longer PFS, and *Parabacteroides* and *Methanobrevibacter* predicted better cancer control (96). Another study found that bacteria-depleted or antibiotic-treated mice transplanted with the fecal microbiota from cancer patients who responded to ICIs showed higher ameliorated antitumor activities to PD-1 inhibitors than mice transplanted with nonresponder feces. Metagenomics of patient fecal samples demonstrated that *Akkermansia muciniphila* levels were associated with clinical benefits to ICIs. Interestingly, administration of *Akkermansia muciniphila* to mice after fecal transfer with nonresponder stools rebounded the anti-PD-1 efficacy, which was attributed to the enhanced migration of CCR9^+^CXCR3^+^CD4^+^ T lymphocytes into the mouse tumor microenvironment in an IL-12-dependent manner (10). Ayelet Sivan et al. found that mice administered *Bifidobacterium* presented enhanced dendritic cell function and concomitant intensified accumulation of CD8(+) T cells in the tumor beds; thus, they alone exhibited antitumor capacity to the same degree as PD-L1 inhibitor, and combination treatment almost eliminated tumor outgrowth (94). These results indicate that manipulating the intestinal flora has the potential to be used as one means to enhance the efficacy of immunotherapy in lung cancer.

Despite the obvious advantages of ICIs, we should not ignore the adverse reactions associated with immunotherapy. Although anti-CTLA 4 and anti-PD-1/PD-L1 antibodies have become the first-line treatment used against a wide variety of tumor types, due to the use of ICIs increasing T cell activity and eliminating the immune system from nature and “braking”, these drugs may be associated with immune-related adverse events (irAEs), especially when used in combination. Studies have issued that the incidence of irAEs, including diarrhea, colitis, fatigue, rash/itching, mucositis and pneumonia, ranges from 15 to 90%, with estimates of severe irAEs ranging from 0.5 to 13% (97). Yun-bin Zhang et al. found that the mechanism of tuberculosis induced by anti-PD-1 treatment may be related to a hypersensitivity response similar to immune reconstructive inflammatory syndrome (IRIS). Pembrolizumab induced substantial CD38 expression in Th17 cells and significantly increased intestinal microbiota diversity in response to the pembrolizumab treatment cycle, suggesting that pembrolizumab may trigger Th17-phenotypic airway inflammation through microbiota interactions along the gut-lung axis (98). Another study examined stool samples taken from 26 patients with advanced lung cancer before they were first given anti-PD-1 antibodies and found that immune-related diarrhea patients were characterized by a lower abundance of *Phascolarctobacterium* belonging to the *Firmicutes* phylum and *Parabacteroides* and *Bacteroides* of the *Bacteroidetes* phylum and a higher abundance of *Veillonella* of the *Proteobacteria* phylum (99).

In addition, researchers suggest that antibiotic-induced imbalance of the gut microbiota may affect the clinical benefit of ICIs in NSCLC patients (100–102). A meta-analysis evaluating the influence of antibiotic use on survival in NSCLC patients treated with PD-1/PD-L1 blockades revealed that patients exposed to antibiotics before ICI treatment had obviously decreased survival, as reflected by the median OS being decreased by an average of 6.7 months (103). However, in some cases, antibiotics have to be used during immunotherapy. How does this affect the efficacy of ICIs? Giulia Galli et al. defined the antibiotic-immunotherapy exposure ratio (AIER) as “days of antibiotic/days of IO” during the whole immunotherapy period (WIOP) and found that NSCLC patients with a higher AIER had shorter PFS and OS than the others (104).

In general, gut microbiota may affect the efficacy of lung cancer immunotherapy and immunorelated toxicity and side effects to a certain extent; thus, artificial intervention of intestinal flora is likely to improve the efficacy and attenuate the toxicity of immunotherapy. In addition, we should be careful to use antibiotics to avoid affecting the efficacy of immunotherapy for lung cancer.

## Probiotics: A Double-Edged Sword

In recent years, probiotics conferring health benefits to hosts have attracted widespread interest. It has been shown that human intervention with probiotics can promote lung health, reduce the severity of lung diseases, and increase resistance to diseases. Remote regulation of pulmonary homeostasis using probiotics underlies the concept of immunomodulatory regulation by beneficial bacteria *via* gut-lung crosstalk. *Lactobacillus plantarum* CIRM653 alleviated the lung inflammatory response in mouse models infected with *Klebsiella pneumoniae* by reducing the counts of lung innate immune cells, such as macrophages and neutrophils, and cytokines, including mouse keratinocyte-derived chemokines TNF-α and IL-6, as well as triggering an immunosuppressive Treg cell response in the lungs (105). In addition, treatment of mice with the recombinant probiotic *Lactobacillus rhamnosus* GR-1 restricted the augmentation of respiratory total cell populations, lymphocyte populations and lung IL-1β levels, thus contributing to lower airway hyperreactivity (19). Other probiotics that regulate immune homeostasis in the lungs, including *Clostridium butyricum* (106), *Lactobacillus rhamnosus* GG (107, 108), *Bifidobacterium longum* (109, 110), *Saccharomyces cerevisiae* UFMG A-905 (111) and *Akkermansia muciniphila* (112), are thought to play a major role in lung health. However, although probiotics are increasingly widely used with outstanding overall security, adverse events remain a potential concern. A study observed that nearly half of the 65 isolated *Bacillus* spp. strains from commercial probiotic products harbor the potential to create hazardous toxins, and mice infected with the representative isolates present with intestinal inflammation, sepsis and liver injury (113). Moreover, multiple antimicrobial resistance genes and mobile genetic elements were sheltered in these strains (113). Overall, *Bacillus* probiotics may have a potential risk for health due to their ability to generate multiple toxins and harbor mobile antimicrobial resistance genes.

Collectively, the ability of probiotics to produce a variety of toxins and carry ambulate antimicrobial resistance genes while enhancing the health of the host indicates a potential health risk and suggests caution in the use of probiotics. A recent study showed that the transient colonization of probiotic strains in the human lower digestive tract is highly variable, with some people allowing colonization and others resisting it. In addition, there were significant individual differences in human pathways affected by these probiotic strains (114). The uncertainty of whether microbes will survive and function in recipients will be removed by using standardized doses of purified microbial ingredients, thus endowing them with stronger potential than probiotics themselves.

## Conclusions and Perspectives

Lung cancer is the leading cause of cancer death worldwide, and smoking, chronic obstructive pulmonary disease (COPD) or emphysema are part of the recognized risk factors for the development of lung cancer. Recent studies have suggested that gut microbiota may influence the susceptibility to malignancy and clinical immunotherapy response. Therefore, we hypothesize that the gut microbiota may be involved in the development of lung cancer and the effectiveness of immunotherapy. Recent efforts to explore the important role of the gut-lung axis in lung disease have also revealed the correlation between specific components of the gut microbiota and their derived metabolites and carcinogenesis and metastasis of lung cancer, as well as lung immunotherapy response. These interactions include gene instability caused by intestinal flora and metabolites, chronic systemic inflammation, disruption of immune homeostasis, and deficiency of immune surveillance. Recent studies have shown that gut microbiota affects the efficacy of ICIs in lung cancer patients and have also demonstrated that probiotics and FMT may be one of the methods to increase the efficacy and reduce the toxicity of immunotherapy. In addition, the use of antibiotics before or during immunotherapy can lead to gut dysbiosis, which in turn affects the efficacy of immunotherapy.

Because of the widespread use of NGS and 16S RNA, most studies have focused on the bacterial components of the gut microbiome. However, the gut microbiome is a large and complex library of microbial signals. The roles of fungi, protozoa, worms, viruses, and bacteriophages may be equally important. Fungi and viruses may also influence pulmonary homeostasis through the gut-lung axis, but little is known about this. In addition, although some studies have shown that certain microorganisms and derivatives can affect and regulate the development of lung cancer and the ICI curative effect, some of the flora were not advantageous bacterial groups. In other words, the content is relatively too small, the effect is still questionable, and separate microbiota may be observed with the change of environment and show different biological function. The present study is only at the qualitative level, and thus more research is necessary to reach a quantitative breakthrough. A better analysis of the association between gut microbiome composition, efficacy and toxicity of antibiotic therapy and ICIs, as well as dynamic monitoring of gut microbiome evolution, can lead to this conclusion and support the use of probiotics or microbial agents to regulate the gut microbiome to improve the prevention and treatment of lung cancer.

## Author Contributions

XJL was mainly responsible for writing conception, literature searching, and drafting of the manuscript. YC participated in the collection of literatures and revision of article. DZ, MZ, XHL and DL were responsible for the revision of the manuscript. BG, HZ, JS, XH and ML provided professional revision for the article. JC participated in the conception and revision of the article. All authors contributed to the article and approved the submitted version.

## Funding

This work was supported by the Chinese Society of Clinical Oncology (CSCO) Research Foundation (Nos. Y-JS2019-034).

## Conflict of Interest

The authors declare that the research was conducted in the absence of any commercial or financial relationships that could be construed as a potential conflict of interest.

## Publisher’s Note

All claims expressed in this article are solely those of the authors and do not necessarily represent those of their affiliated organizations, or those of the publisher, the editors and the reviewers. Any product that may be evaluated in this article, or claim that may be made by its manufacturer, is not guaranteed or endorsed by the publisher.
